# Challenges and facilitators to sexual and reproductive health care for undocumented in-transit migrant women in Mexico: a qualitative study

**DOI:** 10.3389/frph.2025.1683858

**Published:** 2025-09-26

**Authors:** Silvana Larrea-Schiavon, Colette Auerswald, Sylvia Guendelman, Jay Graham, César Infante

**Affiliations:** ^1^ Interdisciplinary Department, School of Public Health, University of California, Berkeley, CA, United States; ^2^ Community Health Sciences, School of Public Health, University of California, Berkeley, CA, United States; ^3^ Wallace Center for Maternal, Child and Adolescent Health, School of Public Health, University of California, Berkeley, CA, United States; ^4^ Division of Environmental Sciences, School of Public Health, University of California, Berkeley, CA, United States; ^5^ Health Systems Research Center, National Institute of Public Health, Cuernavaca, Mexico

**Keywords:** in-transit migration, sexual and reproductive health, policies, Mexico, utilization of healthcare services

## Abstract

**Background:**

The number of international migrants has steadily increased over the past decade. Among them, undocumented in-transit migrant women (UITMW) face heightened vulnerability to gender-based violence and complex sexual and reproductive health (SRH) needs. However, limited evidence exists on the challenges state and non-state actors face in delivering SRH services to this population. This qualitative study explores the barriers encountered by service providers and decision-makers in Mexico when addressing UITMW's social and health needs through an SRH lens, and identifies facilitators that may support more effective service delivery.

**Methods:**

Between August and November 2023, we conducted 31 in-depth interviews with 36 service providers, migration experts, and local and federal decision-makers in Ciudad Juárez, Chihuahua City, and Mexico City. Guided by McLeroy et al.'s socioecological model, we examined challenges and facilitators at individual, institutional, community, and structural levels. Data were analyzed using a framework analysis approach.

**Results:**

Participants identified 11 challenges and 9 facilitators influencing SRH service provision for UITMW. Key challenges included: (1) policies and resource allocations are shaped by the perception of migration as temporary; (2) growing anti-immigrant sentiment undermine community-level service delivery; (3) religious restrictions in faith-based shelters limit access to certain SRH services; and (4) biases among healthcare providers affect quality of care. Notable facilitators included structural reforms such as strengthened migration and health governance and improved multi-level collaboration to enhance service access.

**Conclusion:**

This study underscores the complex, multi-level barriers to delivering SRH care to UITMW in Mexico. Findings point to research and policy priorities, including examining the long-term impacts of migration and health policies on SRH service availability, evaluating alternative delivery models, analyzing the role of media in shaping public opinion, and involving UITMW and local stakeholders in policy development. Addressing these gaps could improve SRH outcomes for UITMW and strengthen the broader health system response for both migrant and local populations.

## 1 Introduction

The global population of international migrants increased sharply between 1990 and 2020, rising from 153 million to 281 million (1). Among the various forms of international mobility, in-transit migration warrants special attention due to the multiple vulnerabilities it entails. In-transit migrants are individuals who spend an unspecified period in one country while en route to a final destination (2). This type of migration is often marked by irregular legal status, unsafe migration routes, and limited access to essential services such as healthcare, shelter, and legal protection (3).

Globally, the rise in migration has been met with increasingly restrictive immigration policies aimed at deterring migrant flows (4). Yet, evidence indicates that such policies do not halt migration, particularly irregular migration (5). Instead, they often render migration routes more dangerous and disrupt migration trajectories, leaving individuals stranded in transit countries with limited access to critical resources such as healthcare, employment, education, and safe housing (6, 7).

Mexico is part of the world's largest migration corridor in terms of the number of people transiting through its territory (1). In 2023, Mexican authorities reported 778,907 events of irregular in-transit migration, 31% referred to women (8). Mexico is also one of the most dangerous transit routes in the region. Migrants in Mexico face violence, disappearances, extortion, arbitrary detentions, and abuses by both criminal organizations and government authorities (9, 10). In 2025, the implementation of increasingly restrictive immigration policies in the United States further constrained legal pathways to entry, leaving thousands of migrants stranded in transit countries like Mexico. As a result, many now remain in legal limbo, with limited options for regular entry to the United States. This has led to a significant increase in both the duration of time spent in transit and the number of asylum applications submitted in Mexico, from 2,137 in 2014 to 78,975 in 2024, with a peak of over 140,000 in 2023 (11, 12).

While the Mexican government has implemented policies to improve access to social services for migrants, including healthcare, reports indicate persistent human rights violations against undocumented in-transit migrants, suggesting that existing policies remain insufficient in safeguarding the economic, social, and cultural rights of this population (13, 14). In response to these gaps in state protection, non-state actors—including non-governmental organizations (NGOs), migrant shelters, and international agencies— play a crucial role in delivering these essential services. However, financial cuts to programs and grants from the United States in 2025 have impacted these organizations, limiting their capacity to provide assistance and leading to the closure of operations by some international agencies and NGOs, as well as the forced shutdown of shelters (12).

Undocumented in-transit migrant women (UITMW) in Mexico face compounded vulnerabilities due to their intersecting gender, migration status, social position, and racial and ethnic identities (15). These intersectional identities increase their risk of gender-based violence and adverse sexual and reproductive health (SRH) outcomes, such as unintended pregnancies and sexually transmitted infections (15–17). Nevertheless, if we adopt the Guttmacher-Lancet Commission's definition of SRH, which frames it as a “state of physical, emotional, mental, and social well-being in relation to all aspects of sexuality and reproduction, not merely the absence of disease, dysfunction, or infirmity”, SRH needs are much broader (18).

A recent article that combined survey data from UITMW with data from interviews with service providers and other stakeholders addressing the health needs of this population found a high prevalence of self-reported SRH needs and a low utilization of SRH services (19). The study aligned with findings from previous studies on in-transit migrant populations (20, 21). It highlighted several individual-level barriers to care experienced by UITMW, examining how time in transit and the presence or absence of instrumental social support influences use of SRH services.

Building on this evidence, the present study situates the SRH of UITMW within the broader, intersecting levels of influence that shape health outcomes throughout the migration journey. These include individual-level determinants related to gender, social position, and knowledge; institutional factors such as service accessibility and funding constraints; community factors including discrimination; and structural determinants such as migration governance and the presence of organized crime.

More specifically, the aim of this qualitative study is to explore, through an SRH lens, the challenges that state and non-state actors in Mexico encounter when addressing the health and social needs of UITMW. It also identifies key facilitators that may support more effective, inclusive, and equitable service delivery. Our findings aim to inform policy and programmatic responses, helping Mexico and other transit countries strengthen their approach to addressing the needs of undocumented in-transit migrants—particularly UITMW—in ways that benefit both migrant and host communities.

## 2 Methods

This qualitative study was conducted primarily in Ciudad Juárez, a border city in the Mexican state of Chihuahua with a longstanding migration history. This study examined the factors influencing UITMW's SRH needs, as well as the challenges and facilitators involved in addressing these needs from the perspectives of service providers, local and federal decision-makers, and migration experts. Since 2018, Ciudad Juárez has experienced a significant increase in undocumented in-transit migration. In 2022, two out of every ten undocumented migrants detained in the United States had crossed the border through Chihuahua (22). This surge led to a rapid expansion of local support services, with the number of migrant shelters growing from two to over 30 between 2018 and 2023, alongside an increased presence of NGOs and international agencies providing care and humanitarian assistance. Although the number of new in-transit migrants arriving in Ciudad Juárez has recently declined—largely due to increasingly restrictive immigration policies implemented by the United States—many undocumented migrants remain stranded in Mexican border cities and other parts of the country. These individuals face a difficult decision: remain in Mexico with an irregular migration status or apply for asylum there, which may limit their future eligibility to seek asylum in the United States.

This study adheres to the Consolidated Criteria for Reporting Qualitative Research (COREQ) checklist (23), which is included in Additional File S1.

### 2.1 Recruitment procedures and study participants

We conducted semi-structured interviews with key informants possessing extensive knowledge of SRH and/or international migration. Participants were based either in Ciudad Juárez or Chihuahua City, where they provided direct services to migrants, or in Mexico City, where they contributed to policy design and implementation at the federal level.

We employed purposive sampling to select key informants who worked in public-sector clinics or hospitals, private-sector services—namely shelters, NGOs, and international agencies—and at different levels of government (federal, state, and municipal) (24). In April 2023, the first author (SL) started to recruit participants through professional connections and word-of-mouth, introducing potential participants to the study via email. SL discussed the study with them to address any questions or concerns before scheduling interviews.

Between August and November 2023, SL conducted 31 interviews with 36 key informants. The final sample included three shelter managers, three healthcare providers from shelters, three healthcare providers from public-sector health services, 15 service providers from NGOs and international agencies, four migration experts, four local decision-makers, and four federal decision-makers. We stopped recruiting participants once thematic saturation was reached and no new insights emerged (25). All invited key informants, except one federal decision-maker, agreed to participate.

### 2.2 Interview guide development and data collection

SL, CI, and CA developed individual interview guides directed at service providers, local decision-makers, federal decision-makers, and migration experts, respectively. Guides were informed by McLeroy et al.'s socioecological model (26), which recognizes that health outcomes are shaped by multiple, intersecting levels of influence, extending beyond individual factors to include interpersonal, community, institutional, and policy-level determinants.

The interview guides explored both challenges and facilitators related to SRH service provision for UITMW. The guides were iteratively refined during data collection to incorporate emergent themes. Interview guides (in English and Spanish) are available in Additional File S2. Core questions, adapted for each stakeholder group, included: (i) What challenges do UITMW face when addressing their SRH needs, including accessing health services?; (ii) What are the main challenges that public-sector health services, shelters, NGOs, and international agencies face in responding to UITMW's SRH needs?; (iii) What strategies have public-sector health services, shelters NGOs, and international agencies recently implemented to mitigate these challenges?; (iv)What contextual, political, and economic factors influence access to SRH services and the design and implementation of relevant laws, policies, programs, or strategies?; (v) What are the main implementation challenges faced by federal and local government institutions?; (vi) What recommendations do you have to improve access to SRH services for UITMW?

SL conducted the interviews via Zoom, over the phone, or in person at the participant's workplace, based on individual preferences. Each interview lasted approximately 90 min and was audio-recorded. Following each interview, SL wrote a post-interview memo. No repeat interviews were conducted. Participants received compensation of $10.00 (∼MXN 200.00) or a keychain of equivalent value.

### 2.3 Data analysis

SL transcribed all interviews verbatim using the Amberscript transcription software (27). SL reviewed, cleaned, and de-identified the transcripts. Audio files and transcripts were stored securely on Box, an encrypted and password-protected online platform.

SL and CI applied Klinberg and colleagues’ framework analysis approach (28) using a combined deductive-inductive process for coding the interviews. The initial codebook was structured around two organizational dimensions: a) level (individual, institutional, community, and structural) and b) type of factor, defined either as a challenge or facilitator.

Additional inductive codes were subsequently introduced to capture the specific nature of the challenges and facilitators described by participants. Examples of inductive codes included: (i) creating community, (ii) resource allocation, (iii) NGOs as intermediaries, (iv) conservative and religious values, and (v) migration as a political factor. The final codebook is available in Additional File S3.

A matrix framework was created in Excel, with participants as rows and codes as columns. SL and CI jointly coded the first two interviews and, in collaboration with CA, refined the initial codebook. SL coded the remaining transcripts, while SL, CI, CA, and SG held regular meetings to review inductive codes and discuss emerging themes. SL summarized relevant content for each cell to support theme development. All data analysis was conducted using the MAXQDA qualitative analysis software (VERBI Software, Berlin, Germany) (29).

Although transcripts were not returned to participants for comments or corrections, in May 2024, a workshop was held to present preliminary findings to participants. Participants affirmed that the results accurately reflected their experiences. No changes were made to the findings following this meeting.

### 2.4 Ethical considerations

This study was approved by the ethics and research committees at the National Institute of Public Health in Mexico (Protocol #1861) and the University of California, Berkeley Committee for the Protection of Human Subjects (CPHS #2023-04-16299). All participants provided written informed consent. To protect confidentiality, participants are identified only by their city, professional role, and sector. We do not report age, gender, or institutional affiliation.

## 3 Results

The number and type of interviews (Zoom/phone or in-person), as well as participant location, age, and gender, are summarized by stakeholder type in Table 1.

**Table 1:** Characteristics of study participants, interview modality, and location (*n* = 31), 2023.

Participant group	Interviews *n* (%)	Participants *n* (%)	Age in years Median (range)	Gender *n* (%)		How interview was conducted *n* (%)		City interview conducted in *n* (%)		
				Women	Men	Zoom/Phone	In-person	CJ	CC	MC
Service providers	19 (61.3)	24 (66.7)	33 (25–62)	19 (79.2)	5 (20.8)	9 (47.4)	10 (52.6)	17 (89.5)	0	2 (10.5)
Local decision makers	4 (12.9)	4 (11.1)	44 (37–52)	2 (50.0)	2 (50.0)	1 (25.0)	3 (75.0)	4 (100)	0	0
Federal decision makers	4 (12.9)	4 (11.1)	40 (35–46)	4 (100.0)	0	4 (100.0)	0	0	0	4 (100)
Migration experts	4 (12.9)	4 (11.1)	40 (33–46)	3 (75.0)	1 (25.0)	4 (100.0)	0	1 (25.0)	1 (25.0)	2 (50.0)
Total	31 (100)	36 (100)	35 (25–62)	28 (77.8)	8 (22.2)	18 (58.1)	13 (41.9)	22 (71.0)	1 (3.2)	8 (25.8)

(1) CJ: Ciudad Juárez; (2) CC: Chihuahua City: (3) MC: Mexico City.

We organized findings on the challenges and facilitators related to SRH service delivery into four levels of influence, based on McLeroy et al.'s socioecological model (26): (i) individual (directly related to the characteristics of UITMW); (ii) institutional (related to the delivery of services in the public-sector and private-sector services); (iii) community (related to the characteristics of the communities through which UITMW are transiting); and, (iv) structural (related to migration and health policies and governance issues affecting UITMW). Illustrative quotes are presented in Table 2, organized by thematic level.

**Table 2:** Challenges and facilitators to using and providing sexual and reproductive (SRH) services to undocumented in-transit migrant women (UITMW) in Mexico. Selected quotes supporting study findings.

Level	Actors	Challenges	Selected quotes	Facilitators	Selected quotes
Individual	UITMW	SRH is not a priority during transit	“Many people now travel with children and their partners and family. But the majority of those who care are women. I mean, we are talking about children under six, seven years old, who require a lot of attention and care […]. We know that care work is very demanding. Often, that makes women put aside everything else, right? Their own health”. *Physician and humanitarian specialist, NGO, Mexico City*	Provide workshops to increase women's SRH knowledge Fostering community among women Strengthening peer networks	“These spaces [NGOs] allow users to get to know each other and create networks. So, I think that's also a plus of providing support because you intervene so that they get to know each other and talk, “Oh, look, she's from Honduras”. “Oh, do you know that eight days ago I saw this person and we went for a talk, very cool”. Because it's also about that, about them believing that there can be networks between them. That they are not alone in these processes that are sometimes complicated”. *Social worker, NGO, Mexico City*
		Low SRH literacy	“Also, these, not myths, but challenges of, “Look, she scratches herself a lot here on her parts and then grabs things without washing her hands. She's going to give me this. How am I going to use the bathroom? What if I get infected?” […] There have even been people who haven't wanted to talk about it, “If the [religious] sisters find out, they're going to discriminate against me. The other women will start saying not to come near me”. The fear of discrimination is very ugly. And even more so with this issue of the place being religious”. *Social worker, Faith-based shelter, Ciudad Juárez*		
Institutional	Public-sector clinics and hospitals	Providers’ biases	“As a doctor, I have had to deal with [migrant] people coming and demanding things from me, when many times my health service doesn't even give medicines to me. Sorry, but it's the truth […]. So, what it is like is that the health personnel and the doctors have this thing of, I unprotect one to care for another. I unprotect those from my house, those from my city, the Tarahumaras who sometimes fight a torrent, to care for another who comes from somewhere else”. *Physician, Public-sector hospital, Ciudad Juárez*	Training of healthcare service providers, especially of physicians	“Maybe they [the physicians] need more training in this part of humanizing. Understand the context and the situation they [the migrants] are going through so that when it comes to even starting a conversation with them, you can avoid phrases like, “Well, why are you here?” *Shelter manager, Secular shelter, Ciudad Juárez*
		Implementation gaps	“Women who come to us to say, “I don't want to continue with the pregnancy”. What we do is channel them, because at the end of the day, we are just a shelter. We channel them to the public health institutions responsible for this, and unfortunately, we have found that the criteria are not defined at the hospital. One time they sent her back to us saying she had to be accompanied by a lawyer”. *Shelter manager, Government-led shelter, Ciudad Juárez* “There are a few guidelines, let's say, that are instructive for those who ultimately operate the services on how to implement that [the policies]. Because, well, I mean, what does it mean to be sensitive to someone's mobility condition? It's not very clear”. *Federal decision-maker, Government institution, Mexico City*	Creating policy implementation guidelines for local health services	“Sometimes the programs are designed at a very high level and not from below, to really know how the institutions are operating and everything. When you hear the government's publications, this and that, and you say, but they [the migrants] keep coming. I mean, don't tell me that it [the migration flow] has decreased because I am seeing it in my day-to-day at the hospital”. *Social worker, Public-sector hospital, Ciudad Juárez*
	Non-governmental organizations and international agencies	Scarce resources and Catholic or Christian donors	“Abortion is not mentioned openly as such because, in fact, it is not like [NGO name] makes it public, right? […] So, in the workshops, the only thing we do is talk about preventing gender violence, we specify what types of violence there are, and we talk, yes, about sexual violence. Many times, women do not talk about it openly [about abortion]; it is only when the workshop is over that they come up and ask […]. But it is not something that [NGO] says openly. Precisely because we also work with donors who are Catholic, Christian”. *Service provider, NGO, Ciudad Juárez*	Stronger involvement of the Mexican government in the response More government oversight of non-governmental shelters	“The Mexican State, from the Federation, must respond because, in reality, all this time, since the MPP program began, it has been very lukewarm in all aspects of migration. It has been like, “Oh, yes, let them wait here in Mexico, and we totally ignore them. We don't know if they have access to health, housing, food, jobs” […]. So, I also think that there is irresponsibility on the part of the Mexican State towards migrants”. *Psychologist, NGO, Ciudad Juárez* “I mean, there are these shelters that are of religious origin, that do have access to public resources, because ultimately they are addressing the problem and, in that sense, by receiving public resources they should be obliged to respect the minimum legal framework of human rights and everything, but they don't do it. And since the State is not interested, well. I mean, eh, these populations [migrants] are not of interest [for the State]. So then they [the shelters] don't do it either”. *Political scientist, NGO and Academia, Chihuahua*
	Migrant shelters	Religious values in faith-based shelters	“The HIV program, which is here at CAPASITS, they go to the shelters, and they have told us that they struggle a lot to distribute condoms. They only, well, eh, give the talks and do the [HIV] rapid tests. But they do tell us a lot about that, because, well, because of orders [to not distribute condoms] from those in charge [of the shelters]”. *Local decision-maker, Government institution, Ciudad Juárez*		
		Lack of training on how to respond to sexual violence cases	“The case [of sexual violence] was discovered, and I was the first person to talk to her, and so I had to activate everything, right? And yes, I have felt… Because it has been difficult, many people are involved, many organizations, a lot of ignorance […]. So, well, like, who needs to know? Who doesn’t? Confidentiality, how far does it go? How many resources do we have to mobilize? Do we give priority to the legal or health? No, we do not know very well. I mean, I feel we are groping in the dark in that sense”. *Nurse volunteer, Faith-based shelter, Ciudad Juárez*		
Community	Mexican population	Anti-immigrant sentiments at the community level, fueled by news in the media and political rhetoric	“In the southeast, well, at least in Tapachula, I did get the impression that the population feels invaded […]. I mean, seeing the streets with a huge migrant population, some working, some not, but there is a perception of threat, of insecurity, of course, of discrimination. And I think that sometimes, one of the challenges for women to seek care is even going out into the street. I mean, I don’t doubt that the population attacks them, or there are conflicts in the community itself”. *Federal decision-maker, Government institution, Mexico City* “But it is a double-edged sword because if you make a specific program for migrants, and, I tell you, there is a whole social demand from other communities and other groups where they say, “Why not us? Why do you make a sexual and reproductive health care program only for migrants?” *Federal decision-maker, Government institution, Mexico City*	No areas of opportunity mentioned at this level	
Structural	Federal government	Migration is conceptualized as a temporary phenomenon, which leads to a lack of investment in resources	*“*I think a lot of the [government] response has been emergent, precisely based on these waves of visibility [the migrant caravans]. One of the most relevant things is that migration is always thought of as something temporary. So, why will I invest in it if it is something temporary? I mean, why am I going to make a specialized or differential public policy if that person is going to pass through? Then why am I going to invest in that person, right? Yes, it is perceived as an exception rather than an everyday issue”. *Federal decision-maker, Government institution, Mexico City*	Strengthening the governance of the Ministry of Health in migration and health issues, including fostering multi-level collaboration	“I think that each state has good practices that they implement. And it would be interesting to talk and learn from each other”. *Local decision-maker, Government institution, Ciudad Juárez* “This population is one whose human rights have been systematically violated. So, the inter-institutional linkage is also fundamental, not only because of the complexity of the structures that have to respond simultaneously but because it is mixed with issues of access to justice”. *Federal decision-maker, Government institution, Mexico City* “It is not a Mexican issue, nor is it a U.S. issue, nor is it a Guatemalan issue. In other words, people are moving from Chile to the United States right now. If these policies remain very local, we expect the impact to be limited to the national territory*”. Researcher, Academia, Mexico City*
		Presence of corruption and lack of mechanisms to monitor and enforce policies at the local level	“The corruption issues are terrible […]. There is no other body, that is, beyond this administrative accountability, there is no legal sanction mechanism, right? There is a very strange loophole in this federal-state transfer, and everything is diluted a lot. I mean, the most you can do is reject the invoices because they did not have sufficient proof, and they [the states] refund it. That's it. And that happens again year after year. And no, there is no solution very close at hand”. *Federal decision-maker, Government institution, Mexico City*		
	Organized crime	Strong presence of organized crime in Mexico, leading to instances of sexual violence and fear of reporting and seeking care among survivors of sexual violence	“Health services sometimes clearly identify security-related issues, but due to the nature of the migration phenomenon, neither the woman nor the person in the clinic has enough confidence in the security entities to report certain things […]. So, the doctor or the nurse realizes that there has been a rape. And what do you do with a woman who has just been raped? Because she is not going to go to the police to file a complaint, organized crime is involved. I mean, what about the doctor? Well, in the end, I mean, what do I do, right? I mean, how do I guide her?” *Federal decision-maker, Government institution, Mexico City*		

The quotes in this table represent a selected sample from the available data. We chose those that best illustrate each challenge or facilitator.

### 3.1 Individual level

At the individual level, interviewees identified two main challenges UITMW face to use SRH services. The first challenge, mentioned by most NGO service providers, is that *SRH is not a priority* for women during their transit. Participants perceive that, given the multiple hardships women experience —such as food and economic insecurity, lack of safe shelter, and exposure to gender-based violence—they often deprioritize their SRH needs. Additionally, most women travel with their children. As the caregiving role falls on them, this has two main impacts on their access to SRH services. First, women prioritize their children and family members' health needs over their own. Second, the absence of childcare in most shelters makes them reluctant to leave their children unattended while seeking SRH services.

The second challenge, emphasized by nearly all interviewees, is that most UITMW have *low SRH literacy*. This includes not only limited knowledge about SRH but also difficulties accessing, understanding, and applying this knowledge to address their SRH needs. For instance, a nurse volunteer at a faith-based shelter noted that many women lack menstrual knowledge that prevents them from recognizing menstrual irregularities that may require medical attention. A social worker in the same shelter observed that low SRH literacy contributes to the spread of misinformation, particularly myths about the risk of contracting sexually transmitted infections from contamination present in shared bathrooms, showers, or utensils. In some cases, these misconceptions have led to discriminatory behaviors within shelters, such as being poorly treated, making women less likely to acknowledge their SRH needs or seek care.

Despite these challenges, several NGO and shelter workers identified key facilitators that could improve SRH service access for UITMW. One key facilitator is the *provision of SRH workshops* within shelters, which many NGOs are already implementing. Expanding the number and focus of these programs (e.g., where to seek care for sexual violence and abortion services) could increase women's knowledge about SRH and where to seek care, ultimately improving service utilization. Another proposed facilitator is *fostering a stronger sense of community among UITMW*, which could empower women and develop strong networks of support that may enable women to seek SRH services. Strengthening peer networks may also encourage care-seeking among women with children, as women who have stronger network ties may be more likely to entrust their children to the care of other shelter residents while they attend medical appointments. Additionally, fostering connections among UITMW can encourage knowledge sharing, helping women learn from one another about managing their SRH needs and navigating available services.

### 3.2 Institutional level: public-sector clinics and hospitals

Beyond increasing UITMW's SRH literacy and strengthening their social support networks, public-sector clinics and hospitals also need to deliver services that are provided in a bias and stigma-free environment. Participants identified several challenges at the public-sector health service level that not only reduce the quality of care but, in some cases, also lead to the denial of care. One key challenge, mentioned by federal decision-makers and public-sector service providers, is *the presence of biases among physicians and other healthcare providers* against migrant populations. A common perception among healthcare providers is that whether UITMW are “good” or “bad” patients often depends on their countries of origin. For example, during interviews, some providers described Cuban and Haitian women as “good patients” because they are perceived as calm, well-groomed, and respectful during medical visits. In contrast, Venezuelan women were described as “bad patients” for voicing complaints, speaking loudly, and treating staff with perceived disrespect.

Interviewees also recount that physicians working in resource-limited settings often experience a moral struggle when allocating care. In public-sector clinics and hospitals, where resources are scarce, some physicians feel their primary duty is to serve local communities first before extending care to migrant populations. As one physician emphasized, the ethical dilemma that physicians face when using limited resources to treat migrant communities instead of serving their local population often involves difficult decisions.

A second challenge, felt by healthcare providers and local decision-makers, is the *implementation gaps* between federal policy mandates and the capacity of local public-sector health services to apply them effectively. Interviewees cited three key factors driving these gaps: (i) the absence of specific guidance regarding how to implement federal migration and health policies and programs; (ii) contradictions between federal and local policies; and, (iii) insufficient training for service providers in the implementation of these policies. Two examples shared during the interviews illustrate this gap. First, a physician in a public-sector hospital explained that while federal policy mandates that migrants must receive care regardless of their documentation status, the health insurer requires physicians to register the services they provide for the state to be reimbursed. However, this registration process requires an official ID, which many migrants do not have. The second example, shared by a federal decision-maker, concerns abortion access in Mexico. Two documents regulate abortion care in Mexico: the Mexican Official Standard 046 (NOM-046) (30) and the Technical Guidelines for Safe Abortion Care in Mexico (31). Both documents clearly state that survivors of sexual violence are not required to file a formal complaint or be accompanied by a lawyer to access abortion services. Additionally, they affirm that healthcare providers who perform abortions following these guidelines cannot be prosecuted. However, interviewees noted that many service providers lack training on abortion rights, leading to misconceptions and fear of legal repercussions, especially when providing abortion care outside of sexual violence cases. A shelter manager further reinforced this issue, describing instances where women were asked to return with a lawyer to access abortion care, thereby adding unnecessary barriers to care.

One key facilitator identified, particularly by NGO and shelter service providers, is the need to *strengthen physician training* in public-sector clinics and hospitals. Interviewees emphasized that healthcare providers should develop stronger competencies to better understand the migration journey and its impact on UITMW's health and rights. A second facilitator, highlighted by federal and local decision-makers as well as public-sector service providers, is the *development of evidence-based guidelines* with a step-by-step approach for improving care of migrant populations. Additionally, local decision-makers and frontline service providers stressed the importance of being involved in the design phase of these policies and guidelines. Their participation would help ensure that policies are adapted to local contexts and address the specific barriers they encounter in practice.

### 3.3 Institutional level: private-sector services

In addition to public sector services provided by clinics and hospitals, non-state actors such as NGOs, international agencies, and migrant shelters also play a key role in providing SRH services to UITMW. However, accessing SRH care through these organizations presents its own set of challenges. A primary challenge identified by NGO and international agencies providers is the *scarcity of resources*, partly due to donors shifting their focus away from Mexico to other countries they perceive as having greater need. Some of the remaining donors, particularly those affiliated with Catholic or Christian communities, prohibit the use of their funds for abortion and post-abortion care. As a result, some NGOs, despite having resources, are hesitant to provide these services, fearing the loss of donor support.

Faith-based shelters, which make up most migrant shelters in Mexico, present additional barriers to SRH care. Interviewees shared that, while most shelters facilitate access to some SRH services (e.g., HIV testing, access to menstrual health products, prenatal and postnatal care, and screening for breast and cervical cancer), their *religious values* often restrict access to services such as birth control, emergency contraception, and abortion care. A local decision-maker shared that health authorities have faced resistance when trying to distribute condoms or provide information about birth control in shelters. Similarly, a physician and a nurse practitioner working in several shelter-based clinics reported instances in which shelter managers advised them against asking women about their contraceptive needs or distributing birth control methods. In addition, many *shelter-based service providers lack training* on how to respond to cases of sexual violence. While shelters have established referral mechanisms to improve health care access to address other scenarios, they have not consistently done so for sexual violence, delaying the identification, referral, and treatment of sexual violence survivors.

Given these challenges, interviewees working in NGOs strongly emphasized the need for *increased federal and local government involvement* in the response, particularly as donors continue to withdraw from Mexico. Other service providers stressed the importance of real-time, reliable data on migration flows and health needs to improve planning, adaptation, and response efforts. Others advocated for *greater government oversight of non-state shelters*, especially in the case of faith-based shelters. They added that additional investments were needed in secular and/or government-led shelters. According to participants, the availability of such shelters would ensure better monitoring and compliance with policies that uphold migrants' rights (e.g., housing, employment, and education), as well as SRH rights (e.g., access to free and quality SRH services, free exercise of sexuality, and reproductive freedom). However, some interviewees expressed concerns about distrust between shelters, NGOs, and the government, noting that this hinders collaboration and coordination efforts. Others cautioned against expanding government oversight, citing past experiences of corruption and abuse of power by authorities.

### 3.4 Community level

The communities through which UITMW transit also play a role in shaping both the response to their SRH needs and their ability to use SRH services. Local and federal decision-makers interviewed noted an *increase in anti-immigrant sentiments* among the Mexican population, particularly in states that have not traditionally received migrants. Interviewees attributed this increase to media narratives and the political rhetoric of federal and local politicians, which they believe have fueled negative perceptions about migrants. They described two main consequences of these anti-immigrant sentiments on SRH provision and utilization. First, they perceived that UITMW increasingly fear leaving shelters to seek SRH care, as they worry about experiencing violence or hostility from local community members. Second, community members have expressed dissatisfaction with the government, perceiving that it is allocating more resources to migrant healthcare needs than to those of the Mexican population. A federal decision-maker shared that these complaints had influenced the government response to migration in Mexico. For example, to avoid further backlash, the government has steered away from targeted approaches, such as mobile clinics or vaccination campaigns specifically for undocumented in-transit migrants. Instead, it has opted for broader, horizontal responses that address the health needs of multiple vulnerable groups, including Indigenous communities, people experiencing homelessness, and migrants. However, as mentioned by federal decision-makers and public-sector health service providers, this horizontal approach has not effectively reduced barriers to SRH care for UITMW, partially because of the lack of guidance on how to address the specific challenges that each of these vulnerable groups face. Interviewees did not share areas of opportunity for the challenges experienced at this level.

### 3.5 Structural level

Migration governance and immigration and health policies are the backdrops for SRH care provision for UITMW, shaping national priorities and responses that either facilitate or hinder access to services. Notably, all participants agreed that non-state actors are the primary providers of healthcare for UITMW, including SRH services. Federal decision-makers offered two key reasons why the government has not allocated more resources to public-sector clinics and hospitals. First, they argued that *migrants are perceived as temporary “residents”* just passing through. Thus, these federal decision-makers report that government actors are not motivated to allocate time, human resources, and money to a population that spends a few days in Mexico. However, NGO and shelter-based service providers contradicted this perception, noting that due to increasing barriers to crossing into the United States, UITMW are now spending much longer periods in Mexico than before. The second reason, cited by federal decision-makers, is the *government's limited capacity to monitor and enforce policy implementation* and resource allocation at the local level. This lack of oversight has led to corruption and implementation gaps. Notably, federal decision-makers attributed implementation gaps to corruption and the mismanagement of existing resources, while local decision-makers and service providers highlighted a genuine lack of resources as the root cause.

A final challenge is related to the strong *presence of organized crime* in Mexico and the collusion of these groups with government authorities, which significantly impacts UITMW's SRH in two main ways. First, it increases women's risk of experiencing sexual violence while in transit. In fact, participants agreed that sexual violence is the main risk women face while migrating through the country. Second, participants shared that fear of retaliation prevents women from reporting sexual violence or seeking care, especially when the perpetrators are linked to organized crime. Other participants noted that women are aware of the ties between organized crime and government actors, leading them to believe that these groups “have eyes everywhere”. Similarly, service providers also fear the consequences of reporting cases of sexual violence linked to organized crime.

Based on these challenges, participants proposed two key areas of action: (1) strengthening federal government governance and (2) fostering collaboration across relevant actors. *Strengthening governance* is considered a key strategy to facilitate an orderly and safe migration (32). Interviewees shared several principles that align with the *Migration Governance Framework* proposed by the International Organization for Migration (32): (1) the government must adhere to international standards and fulfill migrants' rights; (2) the policies should be evidence-based and formulated using a “whole-of-government” approach; and, (3) as stated above, effective governance requires engagement with multiple actors.

Interviewees were especially adamant about the importance of *collaboration and engagement across multiple actors*. Cooperation among non-state actors and between non-state and state actors was seen as critical to avoiding duplication of efforts and designing a more efficient response. One example cited was an inter-agency working group in Ciudad Juárez, created by the State Population Council and the local health jurisdiction, in collaboration with NGOs and international agencies. This initiative has improved care coordination, leading to greater access to health services for migrant populations. Multi-level collaboration was also perceived as beneficial. Local government cooperation was proposed as a way for state-level actors to share best practices and learn from one another. Federal-level collaboration was identified as an area of opportunity, as greater coordination among government entities is crucial for upholding the rights of migrants. Finally, a researcher with over 20 years of experience in migration and health emphasized the need for international collaboration, arguing that country-level approaches alone have proven inadequate in addressing the complexities of migration in the region.

## 4 Discussion

This study examined the multi-level challenges and facilitators influencing state and non-state actors' ability to meet the SRH needs of UITMW in Mexico. Our main contributions to the literature, derived from the data analysis, highlight key challenges and facilitators with direct implications for local and federal policy and decision-making. Specifically, they include: (i) the impact of framing in-transit migration as a temporary vs. a long-term event on policy design and resource allocation, and its consequences for state and non-state actors' ability to provide SRH services for UITMW; (ii) the rise of anti-immigrant sentiments within Mexican communities and its implications for service delivery; (iii) the role that faith-based shelters play in hindering access to some SRH services; (iv) the role of healthcare provider biases in shaping care quality; and, (v) the potential contribution of structural facilitators, such as strengthening migration and health governance and multi-level collaboration, in informing policy and guiding both state and non-state actors to enhance SRH service delivery for UITMW. Below, we discuss each of these thematic findings in detail, beginning with how framing in-transit migration as temporary versus long-term shapes different aspects of service delivery.

Framing in-transit migration as temporary has shaped migration discourse and policy in Mexico, reinforcing the narrative of migration as a crisis or set of crises, a view echoed in political and media discourse across the Global North (33). According to Cantat et al. (34), the “crisis” framing is rooted in the perception of migration as massive and short-term (34). Such framing carries tangible consequences: it encourages short-term, reactive policy responses while neglecting long-term solutions that address structural drivers of vulnerability, such as the lack of regular migration pathways, poverty, and gender-based violence (33, 34). Our findings challenge the continued use of this framing in the Mexican context. More restrictive immigration policies in the United States and Mexico are forcing UITMW to remain in Mexico for longer periods (22). Moreover, despite migration increases in Mexico, undocumented in-transit migrants still represent less than 1% of Mexico's total population (8, 35). This suggests, as shared by some of our interviewees, that migration should no longer be viewed as a temporary crisis but as an ongoing structural condition that requires sustainable, long-term policy responses that address the evolving migration landscape.

Multiple stakeholders also expressed that political discourse, media portrayals, and “crisis” framing of migration have contributed to a rise in anti-immigrant sentiments among the Mexican population. The rising anti-immigrant sentiment was identified by interviewees as a barrier to targeted SRH service delivery for UITMW. This finding aligns with an OXFAM (2023) study showing that over half of Mexicans view migration negatively, driven by concerns about job competition, public safety, and resource scarcity (36). This dynamic mirrors global trends. Kapelner (4) argues that the anti-immigrant backlash seen across multiple countries can contribute to democratic dysfunction and stresses the need for evidence-based frameworks to assess policy responses that balance citizen concerns with migrant rights, preventing unjust exclusionary policies (4). In the health sector, Morey (37) argues that migration and health policies shaped by anti-immigration sentiments exacerbate health disparities through multilevel discrimination, deportation and detention practices, and restricted access to health resources (37). In Mexico, these attitudes have influenced policymaking. Interviewees reported that federal and local governments are reluctant to develop targeted responses—such as mobile clinics or migrant-specific vaccination campaigns—for fear of public backlash. Instead, governments have favored horizontal approaches that include migrants among other vulnerable groups (38). While well-intentioned, such strategies have failed to address the specific needs of UITMW, especially in SRH, highlighting the need for policy approaches that both prioritize the health and rights of migrants and mitigate social tensions.

Even though the number of UITMW may fluctuate depending on policy changes, migration remains a long-term issue requiring sustained efforts. However, the framing of migration as a temporary phenomenon has directly influenced government resource allocation. As one federal decision-maker stated in their interview, why should the government allocate resources to a situation that is temporary? This perspective has led to persistent service gaps, which have been filled by non-state actors, such as NGOS, and migrant shelters, positioning them as key facilitators of health services for undocumented in-transit migrants in Mexico. Nonetheless, our findings highlight the double-edged role of faith-based shelters. On one hand, they provide essential support in a context where state provision is limited. On the other, their religious affiliations often restrict access to comprehensive SRH services. Interviewees reported that some shelter managers discouraged contraception distribution or SRH-related questioning, and that few had protocols for responding to sexual violence. These barriers are particularly concerning given the high levels of gender-based violence that UITMW face during transit. As one of the most accessible points of contact for women in transit, shelters should be a gateway to care—not a gatekeeper. This tension reflects broader conflicts between faith-based humanitarian action and SRH, previously documented in other settings (39). Furthermore, research supports the salience of religion in shaping SRH attitudes and behavior. A United States-based study by Stidham et al. found that women who were religiously observant (attending services weekly and highly valuing religion) had lower SRH service use than their counterparts (40). This is especially relevant in Mexico, where over 80% of women from Central America and 66% from Venezuela—two main regions of origin for UITMW—identify religion as a central part of their lives (41). These findings underscore the need to ensure that migrant women have access to comprehensive, rights-based SRH care, regardless of the religious affiliations of service providers.

When UITMW use public-sector health services, they encounter another key challenge that impacts the quality of care received: the presence of biases among healthcare providers. Interviewees noted that providers often judged UITMW based on their nationality or demeanor, with women from some countries (e.g., Venezuela) stereotyped as more “difficult” or “undeserving” of care. These biases, combined with the perceived pressure to ration limited resources, contributed to the unequal provision of SRH services. This is a notable finding, as most studies in Mexico emphasize the need for provider training on how to better respond to the health needs of undocumented in-transit migrant populations but fail to explicitly acknowledge that such training is necessary to address providers' biases and the specific nature of these biases (42–44). Although we lack direct data on how these biases and discriminatory practices impact UITMW's health outcomes in Mexico, research conducted in the United States by Williams and Mohammed on perceived discrimination and health suggests that discrimination negatively affects healthcare utilization and adherence of underserved populations (e.g., African Americans, Native Americans, and Latino communities), ultimately leading to worse health outcomes (45). Thus, UITMW face a dual barrier—on the one hand, faith-based shelters limit their SRH access due to religious restrictions, and on the other, public-sector clinics and hospitals expose them to discrimination and bias from healthcare providers. Addressing these challenges requires not only increasing provider training on migrant healthcare but also actively combating biases and discriminatory practices that undermine equitable access to SRH services for UITMW.

While migration and health-related challenges seem complex to address, participants identified actionable strategies for improving SRH service delivery that could have positive impacts on both migrant and local communities. At the individual level, fostering peer networks among UITMW was viewed as a powerful way to reduce stigma, increase health literacy, and support care-seeking. Evidence from the United States shows that community-based advocacy and education programs can empower migrant and refugee women, helping them overcome fears, express their needs, and reduce feelings of isolation (46, 47). At the institutional level, interviewees called for more training for providers, the involvement of local actors in policy design, and the development of adaptable guidelines and evidence-based strategies to ensure programs better address their local context and the SRH needs of UITMW. These proposals echo Hudson et al.'s recommendations for closing policy implementation gaps (48). These authors emphasize the importance of designing policies with implementation in mind, tracking progress, providing implementation support, and evaluating outcomes to identify bottlenecks (48). At the structural level, strengthening governance and coordination across sectors emerged as a priority. While non-state actors will continue to play an important role, a more organized, state-led approach is necessary to ensure consistency, accountability, and alignment with human rights standards. Participants emphasized that this shift must include better resource monitoring and regulation of non-state actors, particularly migrant shelters. Implementing a more organized, sustainable, and standardized government response could help alleviate public concerns about resource scarcity, reduce public mistrust, improve service quality, and reduce tensions between migrant and host communities (49).

Even though this study contributes to the growing literature on the challenges and facilitators involved in providing SRH services to UITMW, it has several limitations. The study did not include the voices of UITMW themselves, despite initial plans to do so. Although SL built rapport with migrant women in the shelter where she stayed during fieldwork, after further discussions among the research team we decided that conducting interviews would not be ethically appropriate due to the risk of re-traumatization, as the shelter served women who had recently experienced gender-based violence. Efforts to engage other shelters were unsuccessful, as managers cited concerns about previous “helicopter research”, in which outside researchers collect data in underserved settings without equitable collaboration or tangible benefits for participants (50). Nonetheless, the perspectives of diverse key actors, many with extensive experience working directly with this population, provide valuable insights into the SRH needs of UITMW and the gaps in the protection of their rights. Another limitation is that the research focused exclusively on cisgender women, excluding the experiences of LGBTQ+ migrants who are also at high risk of sexual and gender-based violence and face significant barriers to care. In addition, as a qualitative study using purposive sampling, the findings are not intended to be generalizable to all actors involved in migration and health in Mexico, and the study did not include providers from private or pharmacy-adjacent clinics, which are commonly used for SRH services. Furthermore, while we identified multi-level factors that hinder or facilitate SRH service delivery, the study did not systematically examine how these factors interact with one another; future research should explore these interrelationships more explicitly to better understand the mechanisms shaping SRH access for UITMW. Finally, interviews were conducted both in person and via Zoom or phone call. Although we did not observe noticeable differences in the quality or depth of the information provided, prior studies suggest that interview mode can influence participants' responses (51). We acknowledge this possibility, while also noting that conducting some interviews remotely was necessary to maximize the participation within the available resources and timeframe.

## 5 Conclusions

This study highlights the complex, multi-level barriers to delivering SRH services to UITMW in Mexico. These challenges span individual, institutional, community, and structural domains, and are shaped by intersecting factors such as gender, migration status, discrimination, restrictive immigration policies, and governance gaps. Addressing these barriers is essential for improving access to care and upholding the SRH rights of UITMW. Doing so can also strengthen health systems and benefit both migrant and host communities. Building on our findings, we identify several priority areas for future research that can inform evidence-based policy and programming. First, assess the long-term impacts of migration and health policies on SRH service availability and utilization among UITMW, particularly as immigration policies become more restrictive, this population becomes more diverse, and their length of stay in Mexico increases. Adopting a life course lens to examine the SRH needs of UITMW could also help clarify how barriers and facilitators operate over time and inform the design of preventative and targeted interventions. However, generating this evidence requires timely, reliable data on SRH needs and service access. Second, evaluate alternative SRH service delivery models tailored to UITMW, including their feasibility, accessibility, and effectiveness. These evaluation should also examine whether inclusive models help mitigate public resistance to migrant-focused health services. Third, investigate the role of public discourse and media in shaping public perceptions of UITMW, particularly how narratives portraying them as “burdens” to the healthcare system and other social services may influence attitudes and policy responses. Understanding these dynamics could support the development of communication strategies to combat misinformation and foster more supportive public attitudes. Finally, identify mechanisms to involve public actors in policy design and implementation, as well as how to monitor and enforce policy at the local level. Addressing these priority areas could support a more sustainable, coordinate, and equitable response to the SRH needs of UITMW in Mexico and serve as a model for other transit countries facing similar challenges.

## Data Availability

The datasets presented in this article are not readily availably due to confidentiality concerns and ongoing publications. However, a summary of the data (final matrix) may be made available from the first author (SL) upon reasonable request, subject to approval and in accordance with ethical guidelines. Requests to access the datasets should be directed to Silvana Larrea-Schiavon, silvana.lschiavon@gmail.com.
