# Supplementary material for: Challenges and facilitators to sexual and reproductive health care for undocumented in-transit migrant women in Mexico: a qualitative study
Source: Front Reprod Health. 2025 Sep 26;7:1683858. doi: 10.3389/frph.2025.1683858 (PMC12511078; doi:10.3389/frph.2025.1683858)
Supplement: Supplementary file 2 [file Supplementaryfile2.docx]

Supplementary Material: Additional File 2. Interview Guides in English and Spanish for Service Providers, Local and Federal Decision Makers, and Migration Experts

**Interview Guide**

***Access to sexual and reproductive health services among in-transit migrant women in Mexico: Challenges and opportunities from a systems perspective***

*[Service Providers]*

**To be completed by the interviewer:**

| **Sociodemographic survey** | |
| --- | --- |
| Age: | Gender: |
| Educational level: | Type of service provider: migrant shelter manager, physician, psychologist, social worker, other |
| Organization/Institution: Migrant shelter / CSO / International organization / Pharmacy / Public-sector primary health service / Public-sector hospital / Sanitary Jurisdiction | Time working on migration and health topics: |

**Introduction**

Hello and welcome. We want to thank you for being here today. We greatly appreciate your willingness to share your thoughts, experiences, and opinions with us.

My name is [INSERT NAME]. This interview is part of a study conducted by the University of California, Berkeley and the National Institute of Public Health in Mexico. Women who are migrating through Mexico intending to reach the United States might experience specific health needs during this journey. However, the services provided to them to resolve these needs might be difficult to access. We are conducting this study to learn from service providers, such as yourself, and decision-makers how we can increase access to sexual and reproductive health services for women who are migrating through Mexico. What we are interested in is learning about your experiences providing sexual and reproductive health services to migrant women while they are in Mexico and what are some of the challenges and opportunities they face and the health system face to provide these health services.

The interview will last between 1 and 1.5 hours. Here is a document, called a research consent document, that you can keep. In this document, you will find more information about the aim of the research, the research procedures (what participating in the research looks like), the risks and benefits of the research, and compensation. We will take a moment to read together the consent form. We want to reiterate that your participation is voluntary, which means that you can choose if you want to participate or not.

**[STOP AND READ THE CONSENT FORM WITH THE PARTICIPANT]**

Do you have any questions regarding the Consent Form? [*Answer questions posed by the participant*].

**[IF THE PARTICIPANT DOES NOT CONSENT TO PARTICIPATE STOP THE INTERVIEW AND THANK THEM FOR THEIR TIME].**

I will take notes during the interview. I will also audio record the interview so I can listen to it and improve my notes. The recording will only be used to make sure we have captured everything you share with us. The recording will not be shared, and we will destroy it once we have completed our notes and transcribed it into writing.

Do you have any further questions about the research or your participation in it? [*Answer questions*].

Okay, let´s get started.

**Introductory questions:**

- - - 1. Can you tell me a little bit about your professional background? (Probes: How long have you worked here? What is your role? What is your academic training?)
      2. Can you expand a little on your work with in-transit migrant women? What has been your experience caring for the SRH needs of these women?
      3. What are your perspectives on the SRH of in-transit migrant women and the role of the state in providing care for these needs?

**Sexual and reproductive health needs:**

- - - 1. Based on your experience, what are the main health needs of in-transit migrant women? (Probe if there is no mention of SRH needs: What are the main SRH needs of in-transit migrant women?)

**Access and use of sexual and reproductive health services:**

Challenges and opportunities at the individual level:

- - - 1. Based on your experience, how do in-transit migrant women resolve their SRH needs?
         1. Where do they go to solve their SRH needs? (Note: The answer to this question might vary according to the SRH need. Follow-up questions should include each of these spaces separately. Places: migrant shelters, NGOs/CSOs, international organizations, pharmacy-based clinics, public-sector health services, and private health services).
    1. Questions for the places mentioned in the answer: Why do you think they go here first? (Probe: What makes this place the first option for women?).
    2. Questions for the places not mentioned in the answer: Why do you think they do not go to [specific health service] to solve their SRH needs?

1. What are some of the challenges in-transit migrant women face when trying to solve their SRH needs (seeking and accessing care)?

Challenges and opportunities at the organizational level:

1. Based on your experience, who are the institutions and organizations that are responding to the SRH needs of in-transit migrant women in Mexico, particularly in Ciudad Juárez? (Probes: Are public-sector institutions meeting those needs? Are migrant shelters? Are CSOs? Are international organizations?)
   1. What are the communication and collaboration practices between these organizations? (Probe: Do they collaborate? How? How do they communicate with each other? Are some organizations not working with other groups? Why do you think this is happening?)
   2. What are the communication and collaboration practices, specifically between the not-for-profit sector and the public health sector? (Probe: Do they communicate with each other? How does this communication happen? Why are they not collaborating?)
   3. How can the communication and collaboration practices between the different organizations and institutions working towards providing SRH care for migrant women be improved? OR What are the areas of opportunity that you see in the communication and collaboration practices between the different organizations and institutions working towards providing SRH care for migrant women?
2. Are there SRH needs that are currently not being met by these organizations and institutions? Why do you think these needs are not being met?
3. What are the main challenges public-sector health services face to respond to the SRH needs of in-transit migrant women? (Note: Ask these questions for each group of services providing care).
   1. Are there strategies that have been implemented recently to reduce some of these challenges? Can you tell me a little more about them (Probe: Who implemented it? Has it worked? In what sense? For whom?)
   2. What are public-health services doing well to address these needs? What could be improved? What is missing?

*Macro-level:*

1. What other non-health-related factors impact access to SRH services for in-transit migrant women in Ciudad Juárez? (Probe: social, economic, and political context)
   1. If policies are not mentioned as one of the factors: Based on your experience, what are the key local policies that either facilitate or hinder access to SRH services for in-transit migrant women in Ciudad Juárez?
2. Are there any strategies/programs/interventions being implemented to increase access to SRH services in Ciudad Juárez? Can you tell me a little bit more about these strategies?

**Closing questions:**

1. Do you have any recommendations on how to improve access to SRH services for in-transit migrant women in Ciudad Juárez?
2. What recommendations do you have at the policy level to improve access to SRH services for in-transit migrant women in Ciudad Juárez?
3. Is there anything I should have asked, and I did not, but that you would like to share with me?
4. Is there anything you want to ask me?

**Guía de entrevista**

***Acceso a servicios de salud sexual y reproductiva entre mujeres migrantes en tránsito en México: Retos y oportunidades desde una perspectiva sistémica***

*[Proveedores* *de servicios]*

**A completar por el entrevistador:**

| **Encuesta sociodemográfica** | |
| --- | --- |
| Edad: | Género: |
| Nivel educativo: | Tipo de proveedor de servicios: administrador de refugio para migrantes, médico, psicólogo, trabajador social, otro |
| Organización/Institución: Albergue para migrantes / OSC / Organización internacional / Farmacia / Servicio de salud primaria del sector público / Hospital del sector público / Jurisdicción sanitaria | Tiempo trabajando en temas de migración y salud: |

**Introducción**

Hola y bienvenido/a. Queremos darles las gracias por estar aquí hoy. Apreciamos enormemente su disposición a compartir sus pensamientos, experiencias y opiniones con nosotros.

Mi nombre es [INCLUIR NOMBRE]. Esta entrevista es parte de un estudio realizado por la Universidad de California, Berkeley y el Instituto Nacional de Salud Pública. Las mujeres que están migrando a través de México con el objetivo de llegar a los Estados Unidos, podrían experimentar necesidades específicas de salud durante este viaje. Sin embargo, los servicios que se les proporcionan para resolver estas necesidades pueden ser difíciles de acceder. Estamos llevando a cabo este estudio para aprender de los proveedores de servicios, como usted, y los tomadores de decisiones cómo podemos aumentar el acceso a los servicios de salud sexual y reproductiva para las mujeres que migran a través de México. Lo que nos interesa es conocer sus experiencias brindando servicios de salud sexual y reproductiva a mujeres migrantes mientras están en México y cuáles son algunos de los desafíos y oportunidades que enfrentan y el sistema de salud para brindar estos servicios de salud.

La entrevista durará entre 1 y 1,5 horas. Aquí hay un documento, llamado consentimiento informado, que puede conservar. En este documento encontrará más información sobre el objetivo de la investigación, los procedimientos de investigación (cómo se ve participar en la investigación), los riesgos y beneficios de la investigación y la compensación. Nos tomaremos un momento para leer juntos el formulario de consentimiento. Queremos reiterar que su participación es voluntaria, lo que significa que puede elegir si desea participar o no.

**[DETÉNGASE Y LEA EL FORMULARIO DE CONSENTIMIENTO CON EL PARTICIPANTE]**

¿Tiene alguna pregunta sobre el formulario de consentimiento? [*Responder a las preguntas formuladas por el participante*].

**[SI EL/LA PARTICIPANTE NO DA SU CONSENTIMIENTO PARA PARTICIPAR, DETENGA LA ENTREVISTA Y AGRADÉZCALE POR SU TIEMPO].**

Tomaré notas durante la entrevista. También grabaré la entrevista en audio para poder escucharla y mejorar mis notas. La grabación solo se utilizará para asegurarnos de que hemos capturado todo lo que comparte con nosotros. La grabación no será compartida, y la destruiremos una vez que hayamos completado nuestras notas y la hayamos transcrito por escrito.

¿Tiene más preguntas sobre la investigación o su participación en ella? [*Responder preguntas*].

Bien, comencemos.

**Preguntas introductorias:**

- - - 1. ¿Puede contarme un poco sobre su experiencia profesional? (Probe: ¿Cuánto tiempo ha trabajado aquí? ¿Cuál es su papel? ¿Cuál es su formación académica?)
      2. ¿Puede ampliar un poco su trabajo con mujeres migrantes en tránsito? ¿Cuál ha sido su experiencia atendiendo las necesidades de SSR de estas mujeres?
      3. ¿Cuáles son sus perspectivas sobre la SSR de las mujeres migrantes en tránsito y el papel del Estado en la atención de estas necesidades?

**Necesidades de salud sexual y reproductiva:**

- - - 1. Según su experiencia, ¿cuáles son las principales necesidades de salud de las mujeres migrantes en tránsito? (Investigue si no se mencionan las necesidades de SSR: ¿Cuáles son las principales necesidades de SSR de las mujeres migrantes en tránsito?)

**Acceso y uso de servicios de salud sexual y reproductiva:**

Desafíos y oportunidades a nivel individual:

- - - 1. Según su experiencia, ¿cómo resuelven sus necesidades de SSR las mujeres migrantes en tránsito?
         1. ¿A dónde van para resolver sus necesidades de SSR? (Nota: La respuesta a esta pregunta puede variar según la necesidad de SSR. La pregunta de seguimiento debe incluir cada uno de estos espacios por separado. Lugares: refugios para migrantes, ONG/OSC, organizaciones internacionales, clínicas basadas en farmacias, servicios de salud del sector público y servicios de salud privados).
    1. Preguntas para los lugares mencionados en la respuesta: ¿Por qué considera que las mujeres van aquí primero para atender sus necesidades de SSR? (Probe: ¿Qué hace que este lugar sea la primera opción para las mujeres?).
    2. Preguntas para los lugares no mencionados en la respuesta: ¿Por qué considera que las mujeres migrantes no acuden a [servicio específico de salud] para resolver sus necesidades de SSR?

1. ¿Cuáles son algunos de los desafíos que enfrentan las mujeres migrantes en tránsito cuando intentan resolver sus necesidades de SSR (buscar y acceder a la atención)?

Desafíos y oportunidades a nivel organizacional:

1. Con base en su experiencia, ¿quiénes son las instituciones y organizaciones que están respondiendo a las necesidades de SSR de las mujeres migrantes en tránsito en México, y particularmente en Ciudad Juárez? (Probes: ¿Las instituciones del sector público satisfacen esas necesidades? ¿Son los refugios para migrantes? ¿Son las OSC? ¿Son las organizaciones internacionales?)
   1. ¿Cuáles son las prácticas de comunicación y colaboración entre estas organizaciones? (Probe: ¿Colaboran? ¿Cómo? ¿Cómo se comunican entre sí? ¿Algún grupo de organizaciones no está trabajando con otros grupos? ¿Por qué cree que está sucediendo esto?)
   2. ¿Cuáles son las prácticas de comunicación y colaboración, específicamente entre el sector sin fines de lucro y el sector público? (Probe: ¿Se comunican entre sí? ¿Cómo ocurre esta comunicación? ¿Por qué no están colaborando?)
   3. ¿Cómo se pueden mejorar las prácticas de comunicación y colaboración entre las diferentes organizaciones e instituciones que trabajan para brindar atención de SSR a las mujeres migrantes? O ¿Cuáles son las áreas de oportunidad que ve en las prácticas de comunicación y colaboración entre las diferentes organizaciones e instituciones que trabajan para brindar atención de SSR a las mujeres migrantes?
2. ¿Existen necesidades de SSR que actualmente no están siendo satisfechas por estas organizaciones e instituciones? ¿Por qué considera que estas necesidades no están siendo satisfechas?
3. ¿Cuáles son los principales desafíos que enfrentan los servicios de salud del sector público para responder a las necesidades de SSR de las mujeres migrantes en tránsito? (Nota: Haga estas preguntas para cada grupo de servicios que brindan atención).
   1. ¿Existen estrategias que se hayan implementado recientemente para reducir algunos de estos desafíos? ¿Puede contarme un poco más sobre ellos (Probe: ¿Quién lo implementó? ¿Ha funcionado? ¿En qué sentido? ¿Para quién?)
   2. ¿Qué están haciendo bien los servicios de salud pública para abordar estas necesidades? ¿Qué se podría mejorar? ¿Qué falta por hacer?

*Nivel macro:*

1. ¿Qué otros factores no relacionados con la salud afectan el acceso a los servicios de SSR para las mujeres migrantes en tránsito en Ciudad Juárez? (Probe: contexto social, económico y político)
   1. Si las políticas no se mencionan como uno de los factores: Según su experiencia, ¿cuáles son las políticas locales clave que facilitan o dificultan el acceso a los servicios de SSR para las mujeres migrantes en tránsito en Ciudad Juárez?
2. ¿Existen estrategias/programas/intervenciones que se estén implementando para aumentar el acceso a los servicios de SSR en Ciudad Juárez? ¿Puede contarme un poco más sobre estas estrategias?

**Preguntas finales:**

1. ¿Tiene alguna recomendación sobre cómo mejorar el acceso a los servicios de SSR para las mujeres migrantes en tránsito en Ciudad Juárez?
2. ¿Qué recomendaciones tiene a nivel de políticas para mejorar el acceso a los servicios de SSR para las mujeres migrantes en tránsito en Ciudad Juárez?
3. ¿Hay algo que debería haber preguntado, y no lo hice, pero que le gustaría compartir conmigo?
4. ¿Hay algo que quiera preguntarme?

**Interview Guide**

***Access to sexual and reproductive health services among in-transit migrant women in Mexico: Challenges and opportunities from a systems perspective***

*[Local decision-makers]*

**To be completed by the interviewer:**

| **Sociodemographic survey** | |
| --- | --- |
| Age: | Gender: |
| Educational level: | Professional background: physician, psychologist, social worker, public health, epidemiologist, other |
| Institution: Sanitary Jurisdiction / State Ministry of Health /Other |  |

**Introduction**

Hello and welcome. We want to thank you for being here today. We greatly appreciate your willingness to share your thoughts, experiences, and opinions with us.

My name is [INSERT NAME]. This interview is part of a study conducted by the University of California, Berkeley and the National Institute of Public Health in Mexico. Women who are migrating through Mexico intending to reach the United States might experience specific health needs during this journey. However, the services provided to them to resolve these needs might be difficult to access. We are conducting this study to learn from service providers and decision-makers, such as yourself, how we can increase access to sexual and reproductive health services for women who are migrating through Mexico. What we are interested in is learning about your experiences providing sexual and reproductive health services to migrant women while they are in Mexico and what are some of the challenges and opportunities they face and the health system face to provide these health services.

The interview will last between 1 and 1.5 hours. Here is a document, called a research consent document, that you can keep. In this document, you will find more information about the aim of the research, the research procedures (what participating in the research looks like), the risks and benefits of the research, and compensation. We will take a moment to read together the consent form. We want to reiterate that your participation is voluntary, which means that you can choose if you want to participate or not.

**[STOP AND READ THE CONSENT FORM WITH THE PARTICIPANT]**

Do you have any questions regarding the Consent Form? [*Answer questions posed by the participant*].

**[IF THE PARTICIPANT DOES NOT CONSENT TO PARTICIPATE STOP THE INTERVIEW AND THANK THEM FOR THEIR TIME].**

I will take notes during the interview. I will also audio record the interview so I can listen to it and improve my notes. The recording will only be used to make sure we have captured everything you share with us. The recording will not be shared, and we will destroy it once we have completed our notes and transcribed it into writing.

Do you have any further questions about the research or your participation in it? [*Answer questions*].

Okay, let´s get started.

**Introductory questions:**

- - - 1. Can you tell me a little bit about your professional background? (Probes: How long have you worked here? What is your role? What is your academic training?)
      2. Can you share with me a little bit about your institution? What are your responsibilities within this institution? What has been your experience working with these women?
      3. What are your perspectives on the SRH of in-transit migrant women and the role of the state in providing care for these needs?

**Planning process:**

- - - 1. Can you share with me how programs and strategies regarding the SRH of in-transit migrant women are designed and implemented in Ciudad Juárez or Chihuahua? If no specific SRH program/strategy exists, ask for migration and health programs in general.
         1. What type of information is used to plan and design the programs/strategies?
         2. Who is involved? Who is consulted?
         3. Where do the resources come from? How do you plan for these resources? Who manages these resources?
         4. What are some areas of opportunity regarding this process? How can it be improved?
      2. Based on your experience, what are some of the strategies the *Jurisdicción Sanitaria* and the local health services use to monitor the SRH needs of in-transit migrant women?
         1. What are the areas of opportunity of this strategy? How can it be improved?
      3. How is the information regarding the SRH needs of in-transit migrant women being used in the process of planning the services offered to this population?
         1. If there is no monitoring of SRH needs: How do you plan for the resources needed to offer SRH services to in-transit migrant women?
         2. If there is monitoring, but this information is not being used: Why do you think this information is not used in the planning process?

**Challenges and opportunities of providing sexual and reproductive health services in Ciudad Juárez:**

- - - 1. Based on your experience, who are the institutions and organizations that are responding to the SRH needs of in-transit migrant women in Ciudad Juárez? (Probes: Are public-sector institutions meeting those needs? Are migrant shelters? Are CSOs? Are international organizations?)
  1. How are the *Jurisdicción Sanitaria* and the state MoH communicating and collaborating with each of these institutions?
     1. What is working with these communication and collaboration practices? What is not working and should be improved? What is missing?
  2. How is the *Jurisdicción Sanitaria* communicating and collaborating with the federal institutions to provide SRH care for in-transit migrant women?
     1. What is working with these communication and collaboration practices? What is not working and should be improved? What is missing?

1. What are the main challenges the *Jurisdicción Sanitaria* face to respond to the SRH needs of in-transit migrant women?
   1. Are there strategies that have been implemented recently to reduce some of these challenges? Can you tell me a little more about them (Probe: Has it worked? In what sense? For whom?)
2. What are the main challenges the state Ministry of Health faces to respond to the SRH needs of in-transit migrant women?
   1. Are there strategies that have been implemented recently to reduce some of these challenges? Can you tell me a little more about them (Probe: Has it worked? In what sense? For whom?)
3. What are the main challenges the federal MoH face to respond to the SRH needs of in-transit migrant women?
   1. Are there strategies that have been implemented recently to reduce some of these challenges? Can you tell me a little more about them (Probe: Has it worked? In what sense? For whom?)
4. To the best of your knowledge, and based on your experience, what are current national, state, and local policies that are or could improve access to SRH services for in-transit migrant women?
   1. What are some policies that are or could hinder access to these services? How would you propose to modify these policies to improve access to SRH care for in-transit migrant women?

**Closing questions:**

1. Do you have any additional recommendations on how to improve access to SRH services for in-transit migrant women in Ciudad Juárez? And in Mexico?
2. What additional recommendations do you have at the policy level to improve access to SRH services for in-transit migrant women in Ciudad Juárez? And in Mexico?
3. Is there anything I should have asked, and I did not, but that you would like to share with me?
4. Is there anything you want to ask me?

**Guía de entrevista**

***Acceso a servicios de salud sexual y reproductiva entre mujeres migrantes en tránsito en México: Retos y oportunidades desde una perspectiva sistémica***

*[Tomadores de decisiones locales]*

**A completar por el entrevistador:**

| **Encuesta sociodemográfica** | |
| --- | --- |
| Edad: | Género: |
| Nivel educativo: | Experiencia profesional: médico, psicólogo, trabajador social, salud pública, epidemiólogo, abogado, otros |
| Institución: Jurisdicción Sanitaria / Ministerio de Salud del Estado /Otros |  |

**Introducción**

Hola y bienvenido/a. Queremos darles las gracias por estar aquí hoy. Apreciamos enormemente su disposición a compartir sus pensamientos, experiencias y opiniones con nosotros.

Mi nombre es [INCLUIR NOMBRE]. Esta entrevista es parte de un estudio realizado por la Universidad de California, Berkeley y el Instituto Nacional de Salud Pública. Las mujeres que están migrando a través de México con el objetivo de llegar a los Estados Unidos, podrían experimentar necesidades específicas de salud durante este viaje. Sin embargo, los servicios que se les proporcionan para resolver estas necesidades pueden ser difíciles de acceder. Estamos llevando a cabo este estudio para aprender de los proveedores de servicios y tomadores de decisiones, como usted, cómo podemos aumentar el acceso a los servicios de salud sexual y reproductiva para las mujeres que migran a través de México. Lo que nos interesa es conocer sus experiencias brindando servicios de salud sexual y reproductiva a mujeres migrantes mientras están en México y cuáles son algunos de los desafíos y oportunidades que enfrentan y el sistema de salud para brindar estos servicios de salud.

La entrevista durará entre 1 y 1,5 horas. Aquí hay un documento, llamado consentimiento informado, que puede conservar. En este documento encontrará más información sobre el objetivo de la investigación, los procedimientos de investigación (cómo se ve participar en la investigación), los riesgos y beneficios de la investigación y la compensación. Nos tomaremos un momento para leer juntos el formulario de consentimiento. Queremos reiterar que su participación es voluntaria, lo que significa que puede elegir si desea participar o no.

**[DETÉNGASE Y LEA EL FORMULARIO DE CONSENTIMIENTO CON EL PARTICIPANTE]**

¿Tiene alguna pregunta sobre el formulario de consentimiento? [*Responder a las preguntas formuladas por el participante*].

**[SI EL/LA PARTICIPANTE NO DA SU CONSENTIMIENTO PARA PARTICIPAR, DETENGA LA ENTREVISTA Y AGRADÉZCALE POR SU TIEMPO].**

Tomaré notas durante la entrevista. También grabaré la entrevista en audio para poder escucharla y mejorar mis notas. La grabación solo se utilizará para asegurarnos de que hemos capturado todo lo que comparte con nosotros. La grabación no será compartida, y la destruiremos una vez que hayamos completado nuestras notas y la hayamos transcrito por escrito.

¿Tiene más preguntas sobre la investigación o su participación en ella? [*Responder preguntas*].

Bien, comencemos.

**Preguntas introductorias:**

- - - 1. ¿Puede contarme un poco sobre su experiencia profesional? (Probes: ¿Cuánto tiempo ha trabajado aquí? ¿Cuál es su papel? ¿Cuál es su formación académica?)
      2. ¿Puede compartir conmigo un poco de su institución? ¿Cuáles son sus responsabilidades dentro de esta institución? ¿Cuál ha sido su experiencia trabajando con las mujeres migrantes?
      3. ¿Cuáles son sus perspectivas sobre la SSR de las mujeres migrantes en tránsito y el papel del Estado en la atención de estas necesidades?

**Proceso de planificación:**

- - - 1. ¿Puede compartir conmigo cómo se diseñan e implementan los programas y estrategias relacionados con la SSR de las mujeres migrantes en tránsito en Ciudad Juárez o en el estado de Chihuahua? (Nota: Si no existe un programa/estrategia específica de SSR, preguntar programas de migración y salud en general)
         1. ¿Qué tipo de información se utiliza para planificar y diseñar los programas/estrategias?
         2. ¿Quién está involucrado? ¿A quién se consulta?
         3. ¿De dónde provienen los recursos? ¿Cómo se planifican estos recursos? ¿Quién gestiona estos recursos?
         4. ¿Cuáles son algunas áreas de oportunidad con respecto a este proceso? ¿Cómo se puede mejorar?
      2. Con base en su experiencia, ¿cuáles son algunas de las estrategias que utilizan la *Jurisdicción Sanitaria* y los servicios de salud locales para monitorear las necesidades de SSR de las mujeres migrantes en tránsito?
         1. ¿Cuáles son las áreas de oportunidad de esta estrategia? ¿Cómo se puede mejorar?
      3. ¿Cómo se está utilizando la información sobre las necesidades de SSR de las mujeres migrantes en tránsito en el proceso de planificación de los servicios ofrecidos a esta población?
         1. Si no hay monitoreo de las necesidades de SSR: ¿Cómo se planifican los recursos necesarios para ofrecer servicios de SSR a las mujeres migrantes en tránsito?
         2. Si hay monitoreo, pero esta información no se está utilizando: ¿Por qué cree que esta información no se utiliza en el proceso de planificación?

**Desafíos y oportunidades de brindar servicios de salud sexual y reproductiva en Ciudad Juárez:**

- - - 1. Con base en su experiencia, ¿quiénes son las instituciones y organizaciones que están respondiendo a las necesidades de SSR de las mujeres migrantes en tránsito en Ciudad Juárez? (Probe: ¿Las instituciones del sector público satisfacen esas necesidades? ¿Son los refugios para migrantes? ¿Son las OSC? ¿Son las organizaciones internacionales?)
  1. ¿Cómo se comunican y colaboran la Jurisdicción Sanitaria y las Secretaría de Salud del Estado con cada una de estas instituciones?
     1. ¿Qué funciona con respecto a estas prácticas de comunicación y colaboración? ¿Qué no funciona y debe mejorarse? ¿Qué hace falta?
  2. ¿Cómo se comunican y colaboran la Jurisdicción Sanitaria con las instituciones federales para brindar atención de SSR a las mujeres migrantes en tránsito?
     1. ¿Qué funciona con respecto a estas prácticas de comunicación y colaboración? ¿Qué no funciona y debe mejorarse? ¿Qué hace falta?

1. ¿Cuáles son los principales desafíos que enfrenta la *Jurisdicción Sanitaria* para responder a las necesidades de SSR de las mujeres migrantes en tránsito?
   1. ¿Existen estrategias que se hayan implementado recientemente para reducir algunos de estos desafíos? ¿Puede contarme un poco más sobre ellas (Probe: ¿Han funcionado? ¿En qué sentido? ¿Para quién?)
2. ¿Cuáles son los principales desafíos que enfrenta la Secretaría de Salud del estado para responder a las necesidades de SSR de las mujeres migrantes en tránsito?
   1. ¿Existen estrategias que se hayan implementado recientemente para reducir algunos de estos desafíos? ¿Puede contarme un poco más sobre ellas (Probe: ¿Han funcionado? ¿En qué sentido? ¿Para quién?)
3. ¿Cuáles son los principales desafíos que enfrenta la Secretaría de Salud federal para responder a las necesidades de SSR de las mujeres migrantes en tránsito?
   1. ¿Existen estrategias que se hayan implementado recientemente para reducir algunos de estos desafíos? ¿Puede contarme un poco más sobre ellas (Probe: ¿Han funcionado? ¿En qué sentido? ¿Para quién?)
4. Basándose en su experiencia, ¿cuáles son las políticas nacionales, estatales y locales actuales que están mejorando o que podrían mejorar el acceso a los servicios de SSR para las mujeres migrantes en tránsito?
   1. ¿Cuáles son algunas políticas que están o podrían dificultar el acceso a estos servicios? ¿Cómo propondría modificar estas políticas para mejorar el acceso a la atención de SSR para las mujeres migrantes en tránsito?

**Preguntas finales:**

1. ¿Tiene alguna recomendación adicional sobre cómo mejorar el acceso a los servicios de SSR para las mujeres migrantes en tránsito en Ciudad Juárez? ¿Y en México?
2. ¿Qué recomendaciones adicionales tiene a nivel de políticas para mejorar el acceso a los servicios de SSR para las mujeres migrantes en tránsito en Ciudad Juárez? ¿Y en México?
3. ¿Hay algo que debería haber preguntado, y no lo hice, pero que le gustaría compartir conmigo?
4. ¿Hay algo que quiera preguntarme?

**Interview Guide**

***Access to sexual and reproductive health services among in-transit migrant women in Mexico: Challenges and opportunities from a systems perspective***

*[Federal health decision-makers and migration experts]*

**To be completed by the interviewer:**

| **Sociodemographic survey** | |
| --- | --- |
| Age: | Gender: |
| Educational level: | Professional background: physician, psychologist, social worker, public health, epidemiologist, other |
| Institution: CNEGSR / MoH / IMUMI / Other |  |

**Introduction**

Hello and welcome. We want to thank you for being here today. We greatly appreciate your willingness to share your thoughts, experiences, and opinions with us.

My name is [INSERT NAME]. This interview is part of a study conducted by the University of California, Berkeley and the National Institute of Public Health in Mexico. Women who are migrating through Mexico intending to reach the United States might experience specific health needs during this journey. However, the services provided to them to resolve these needs might be difficult to access. We are conducting this study to learn from service providers and decision-makers, such as yourself, how we can increase access to sexual and reproductive health services for women who are migrating through Mexico. What we are interested in is learning about your experiences providing sexual and reproductive health services to migrant women while they are in Mexico and what are some of the challenges and opportunities they face and the health system face to provide these health services.

The interview will last between 1 and 1.5 hours. Here is a document, called a research consent document, that you can keep. In this document, you will find more information about the aim of the research, the research procedures (what participating in the research looks like), the risks and benefits of the research, and compensation. We will take a moment to read together the consent form. We want to reiterate that your participation is voluntary, which means that you can choose if you want to participate or not.

**[STOP AND READ THE CONSENT FORM WITH THE PARTICIPANT]**

Do you have any questions regarding the Consent Form? [*Answer questions posed by the participant*].

**[IF THE PARTICIPANT DOES NOT CONSENT TO PARTICIPATE STOP THE INTERVIEW AND THANK THEM FOR THEIR TIME].**

I will take notes during the interview. I will also audio record the interview so I can listen to it and improve my notes. The recording will only be used to make sure we have captured everything you share with us. The recording will not be shared, and we will destroy it once we have completed our notes and transcribed it into writing.

Do you have any further questions about the research or your participation in it? [*Answer questions*].

Okay, let´s get started.

**Introductory questions:**

- - - 1. Can you tell me a little bit about your professional background? (Probes: How long have you worked here? What is your role? What is your academic training?)
      2. Can you share with me a little bit about your institution? What is its mandate and how does it relate to the topic of migration and health? What are your responsibilities within this institution?
      3. What are your perspectives on the SRH of in-transit migrant women and the role of the country in providing care for these needs?

**Policies regarding the SRH of in-transit migrant women and the decision-making process:**

- - - 1. What are the policies/laws that are related to providing SRH services for in-transit migrant women in Mexico?

1. Based on your experience and knowledge, what do you think is working with these policies? What are some things that could be improved regarding these policies/laws?
   - - 1. How are the decisions regarding which policies/programs/strategies are needed and how laws should be improved regarding the SRH care of in-transit migrant women being made? Who is included in this decision-making process? What information is included to make these decisions?
2. What do you think is working well regarding this decision-making process? What could be improved? What is missing?
   - - 1. What are the contextual, political, and economic factors that impact the design and implementation of these laws/policies/programs/strategies?
       2. What are the challenges regarding the provision of SRH care for in-transit migrant women that are not being addressed by these policies/laws? What is still missing? Why do you think these challenges have not yet been addressed by the policies and laws?

**Institutions at the federal level:**

- - - 1. What are the institutions, at the federal level, that are involved in the decision-making process and/or provision of SRH care for in-transit migrant women?
      2. What are the collaboration and communication practices between these institutions? And between the federal and state and local levels?

1. What is working well with these collaboration and communication practices? What could be improved? What is still missing?
   - - 1. What are the main challenges federal institutions face regarding implementing the necessary mechanisms to provide SRH care for in-transit migrant women in Mexico?
       2. What are the main challenges state and local institutions face regarding providing SRH care for in-transit migrant women in Mexico?

**Closing questions:**

- - - 1. Do you have any recommendations on how to improve access to SRH services for in-transit migrant women in Mexico?
      2. What recommendations do you have at the policy level to improve access to SRH services for in-transit migrant women in Mexico?
      3. Is there anything I should have asked, and I did not, but that you would like to share with me?
      4. Is there anything you want to ask me?

**Guía de entrevista**

***Acceso a servicios de salud sexual y reproductiva entre mujeres migrantes en tránsito en México: Retos y oportunidades desde una perspectiva sistémica***

*[Responsables federales de la toma de decisiones en materia de salud y personas expertas en salud y migración]*

**A completar por el entrevistador:**

| **Encuesta sociodemográfica** | |
| --- | --- |
| Edad: | Género: |
| Nivel educativo: | Experiencia profesional: médico, psicólogo, trabajador social, salud pública, epidemiólogo, abogado, politólogo, otros |
| Institución: CNEGSR / MoH / IMUMI / Otros |  |

**Introducción**

Hola y bienvenido/a. Queremos darle las gracias por estar aquí hoy. Apreciamos enormemente su disposición a compartir sus pensamientos, experiencias y opiniones con nosotros.

Mi nombre es [INCLUIR NOMBRE]. Esta entrevista es parte de un estudio realizado por la Universidad de California, Berkeley y el Instituto Nacional de Salud Pública. Las mujeres que están migrando a través de México con el objetivo de llegar a los Estados Unidos, podrían experimentar necesidades específicas de salud durante este viaje. Sin embargo, los servicios que se les proporcionan para resolver estas necesidades pueden ser difíciles de acceder. Estamos llevando a cabo este estudio para aprender de los proveedores de servicios y tomadores de decisiones, como usted, cómo podemos aumentar el acceso a los servicios de salud sexual y reproductiva para las mujeres que migran a través de México. Lo que nos interesa es conocer sus experiencias brindando servicios de salud sexual y reproductiva a mujeres migrantes mientras están en México y cuáles son algunos de los desafíos y oportunidades que enfrentan y el sistema de salud para brindar estos servicios de salud.

La entrevista durará entre 1 y 1,5 horas. Aquí hay un documento, llamado consentimiento informado, que puede conservar. En este documento encontrará más información sobre el objetivo de la investigación, los procedimientos de investigación (cómo se ve participar en la investigación), los riesgos y beneficios de la investigación y la compensación. Nos tomaremos un momento para leer juntos el formulario de consentimiento. Queremos reiterar que su participación es voluntaria, lo que significa que puede elegir si desea participar o no.

**[DETÉNGASE Y LEA EL FORMULARIO DE CONSENTIMIENTO CON EL PARTICIPANTE]**

¿Tiene alguna pregunta sobre el formulario de consentimiento? [*Responder a las preguntas formuladas por el participante*].

**[SI EL/LA PARTICIPANTE NO DA SU CONSENTIMIENTO PARA PARTICIPAR, DETENGA LA ENTREVISTA Y AGRADÉZCALE POR SU TIEMPO].**

Tomaré notas durante la entrevista. También grabaré la entrevista en audio para poder escucharla y mejorar mis notas. La grabación solo se utilizará para asegurarnos de que hemos capturado todo lo que comparte con nosotros. La grabación no será compartida, y la destruiremos una vez que hayamos completado nuestras notas y la hayamos transcrito por escrito.

¿Tiene más preguntas sobre la investigación o su participación en ella? [*Responder preguntas*].

Bien, comencemos.

**Preguntas introductorias:**

- - - 1. ¿Puede contarme un poco sobre su experiencia profesional? (Probe: ¿Cuánto tiempo ha trabajado aquí? ¿Cuál es su papel? ¿Cuál es su formación académica?)
      2. ¿Puede compartir conmigo un poco de su institución? ¿Cuál es su mandato y cómo se relaciona con el tema de la migración y la salud? ¿Cuáles son sus responsabilidades dentro de esta institución?
      3. ¿Cuáles son sus perspectivas sobre la SSR de las mujeres migrantes en tránsito y el papel del país en la atención de estas necesidades?

**Políticas relativas a la salud sexual y reproductiva de las mujeres migrantes en tránsito y proceso de toma de decisiones:**

- - - 1. ¿Cuáles son las políticas/leyes relacionadas con la prestación de servicios de SSR para mujeres migrantes en tránsito en México?

1. Con base en su experiencia y conocimiento, ¿qué cree que está funcionando con estas políticas? ¿Cuáles son algunas cosas que podrían mejorarse con respecto a estas políticas / leyes?
   - - 1. ¿Cómo se toman las decisiones con respecto a qué leyes/políticas/programas/estrategias son necesarias para mejorar la atención de SSR a las mujeres migrantes en tránsito? ¿Quién está incluido en este proceso de toma de decisiones? ¿Qué información se incluye para tomar estas decisiones?
2. ¿Qué considera que está funcionando bien con respecto a este proceso de toma de decisiones? ¿Qué se podría mejorar? ¿Qué hace falta?
   - - 1. ¿Cuáles son los factores contextuales, políticos y económicos que afectan el diseño y la implementación de estas leyes/políticas/programas/estrategias?
       2. ¿Cuáles son los desafíos relacionados con la prestación de servicios de SSR para las mujeres migrantes en tránsito que no están siendo abordados por estas políticas / leyes? ¿Qué falta todavía? ¿Por qué cree que estos desafíos aún no han sido abordados por las políticas y las leyes?

**Instituciones a nivel federal:**

- - - 1. ¿Cuáles son las instituciones, a nivel federal, que participan en el proceso de toma de decisiones y/o la prestación de atención de SSR para mujeres migrantes en tránsito?
      2. ¿Cuáles son las prácticas de colaboración y comunicación entre estas instituciones? ¿Y entre los niveles federal, estatal y local?

1. ¿Qué funciona bien con estas prácticas de colaboración y comunicación? ¿Qué se podría mejorar? ¿Qué falta todavía?
   - - 1. ¿Cuáles son los principales retos que enfrentan las instituciones federales en cuanto a la implementación de los mecanismos necesarios para brindar atención de SSR a las mujeres migrantes en tránsito en México?
       2. ¿Cuáles son los principales desafíos que enfrentan las instituciones estatales y locales con respecto a la atención de SSR para mujeres migrantes en tránsito en México?

**Preguntas finales:**

- - - 1. ¿Tiene alguna recomendación sobre cómo mejorar el acceso a los servicios de SSR para las mujeres migrantes en tránsito en México?
      2. ¿Qué recomendaciones tiene a nivel de políticas para mejorar el acceso a los servicios de SSR para las mujeres migrantes en tránsito en México?
      3. ¿Hay algo que debería haber preguntado, y no lo hice, pero que le gustaría compartir conmigo?
      4. ¿Hay algo que quiera preguntarme?
