# Supplementary material for: Challenges and facilitators to sexual and reproductive health care for undocumented in-transit migrant women in Mexico: a qualitative study
Source: Front Reprod Health. 2025 Sep 26;7:1683858. doi: 10.3389/frph.2025.1683858 (PMC12511078; doi:10.3389/frph.2025.1683858)
Supplement: Supplementary file 3 [file Supplementaryfile3.docx]

Supplementary Material. Additional File 3. Final Codebook.

**CODES AND SUBCODES:**

1. ***Participants background and profile***
2. **Professional background**

- Includes information about academic training, work with migrants in general, and work with migrant women specifically.
- Subcode: SRH (work on SRH)
- Examples: Psychologist, working with migrant women since 2020. Main work revolves around offering psychosocial services to the population.

1. **Personal background**

- Includes information about identities of participants interviewed, personal perspectives (e.g., feminist), and other relevant information on participants mental maps that influences how they approach the work they do.
- Example: Participant self-identifies as a feminist researcher. Believes in agency of migrant women and the power and importance of community-level organization.

1. **Organization/Institution background**

- Includes information about mission, focus, programs, target population, and location where the organization/institution does work, as well as services offered by the organization.
- Example: International organization working in Mexico since 2020 and in Ciudad Juárez since 2022. Specializes in preventing and addressing gender-based violence. Programs implemented for migrant women have focus on providing resources to increase their financial stability.

1. ***Individual level***
2. **Barriers and facilitators to care at the individual level**

- Includes information on migrant women regarding:
  - Health literacy, health beliefs, trust and expectations on health needs and health personnel of institutions or organizations, and personal priorities.
  - Personal and social values, culture, autonomy, knowledge, and social networks that facilitate or hinder them from seeking health services.
  - Their living environments, mobility, formal networks, family-related responsibilities that can either facilitate or hinder their ability to reach health services.
  - Income, assets, social capital, and access to health insurance that can either facilitate or hinder their ability to pay and use health services.
  - Agency, information, adherence, caregiver support, and mobility and length of stay that can either facilitate or hinder their ability to engage with health systems.
- Examples: Health is not a priority for migrant women.
- **Subcodes:** facilitator; barrier

1. ***Institutional level***
2. **Barriers and facilitators to care at the institutional level**

- Includes information on health services provided by governmental institutions or CSOs regarding:
  - Transparency, outreach, information, and screening.
  - Professional values, norms, culture, and gender of providers that either facilitate or hinders women from seeking health services there.
  - Geographic location, accommodation, hours of opening, appointment mechanisms, knowledge, and operationalization of rights that can either facilitate or hinder women´s ability to reach health services.
  - Direct and indirect costs of health services, opportunity costs, and resources available for migrants that can either facilitate or hinder women´s ability to use health services.
  - Technical and interpersonal quality, adequacy of services, referral system, and interpreters which can either facilitate or hinder women´s ability to engage with health systems.
- Example: Government health services implement outreach activities in shelters to provide primary health services to the migrant populations (coding system: approachability + outreach + facilitator).
- **Subcodes:** facilitator; barrier

1. ***Community level***
2. **Local policies**

- Includes information on local policies regarding migration and health that are impacting access and use of health services by migrant women.
- Example: Chihuahua is a very conservative state. Abortion care is only available to women if the causal for the abortion is sexual violence (coding system: local policies + barrier).
- **Subcodes:** facilitator; barrier

1. **Programs**

- Includes information on local programs being implemented to respond to the health needs of migrant women.
- Example: One of the PHC centers in Ciudad Juárez was specifically implemented and designed to increase access to PHC services for migrant populations.

1. **Religion**

- Information about how religion or being a religious space hinders or facilitates access to SRH services.

1. **Academia**

- Information around academia and research and how it relates to the topic of migration and health. How academia can positively or negatively impact migrant and other underserved populations.

1. **Local decision-making processes**

- Includes information on how decisions are being made at the local level, as well as who is involved in these decisions.
- Example: Decisions at the local level are mostly based on the political agenda of local decision-makers (coding system: local decision-making processes + barrier).
- **Subcodes:** facilitator; barriers

1. **Communication and collaboration practices**

- Includes information on local communication and collaboration practices that are being implemented at the local level to respond to the health needs of migrant populations.
- Includes information on specifics of this communication and collaboration practices that are either considered as best practices or areas of opportunities.
- Example: organizations and local government institutions have implemented healthcare networks to respond to the health needs of migrant populations (coding system: communication and collaboration practices + best practices).

1. **Sustainability**

- Information about the sustainability of projects/programs or organizations.

1. **Trust in organization/institutions**

- Information about relationships or perceptions of trusts in organizations and/or gov institutions by other organizations.

1. **Local context**

- Includes relevant information about the local context of Ciudad Juárez that is either facilitating or hindering access to health services for migrant women and/or impacting women´s health.
- Includes relevant information on community perceptions of migrant populations in Ciudad Juárez.
- Example: Ciudad Juárez has high levels of gender-based violence and is a known route for human trafficking (coding system: local context + barrier).
- **Subcodes:** facilitator; barrier

1. ***Structural level***
2. **National context**

- Includes relevant information about the national context that is either facilitating or hindering access to health services for migrant women and/or impacting women´s health.
- Example: Political polarization is affecting the implementation of federal programs related to migration and health at the local level (coding system: national context + barrier).
- **Subcodes:** facilitator; barrier

1. **Political drivers**

- Includes information on political factors that impact the provision of health services to migrant populations in Mexico.
- Includes information on national public policies that either facilitate or hinder access and use of health services for migrant women.
- Includes information on federal programs that are being implemented to facilitate access to health services for migrant women.
- Example: The United States dictate migration policies that directly affects how Mexico responds to the migration flows (political drivers + barrier).
- **Subcodes:** facilitator; barrier

1. **Federal decision-making processes**

- Includes information on how decisions are being made at the federal level, as well as who is involved in these decisions.
- Example: Migrant communities are not part of the decision-making process (coding system: federal decision-making process + barrier).
- **Subcodes:** facilitator; barrier

1. **Implementation mechanisms**

- Includes information on if and how the federal programs and instructions are being implemented at the local level.
- Includes strategies related to the implementation of federal programs at the local level.
- Example: You need a key person at the local level that has the political will to implement the program (coding system: implementation mechanisms + facilitator).
- **Subcodes:**  facilitator; barrier

1. **Economic and social drivers**

- Includes information on economic and social drivers that are either facilitating of hindering access and use of health services by migrant women.
- Example: There was a restructuring on how cancer medications were bought at the federal level, and now there is a shortage of medications in the country (coding system: economic and social drivers + barrier).
- **Subcodes:** facilitator; barrier

1. **Lenses**

- Includes information on how the use of different lenses to approach the migration phenomena either facilitating of hindering access and use of health services by migrant women.
- Examples: national security lens vs. human rights lens.

1. **Other structural drivers**

- Other drivers at the structural level
- **Subcodes:** Institutional violence; predictability; wear; data; facilitator; barrier

1. ***Other codes***
2. **Gender**

- Includes any information about differences in exposure, health needs, and access and use of health services, amongst other, between migrant women, men, and LGBTQ+ migrant populations.
- Examples: Women are more exposed than men to sexual abuse and violence during transit. Women create more community with other women, creating informal networks, when compared to men.
- **Subcodes:** facilitator; barrier

1. **Migration as determinant**

- Includes any information about the added layer of vulnerability migrant women face, due to their immigration status, when transiting through the country and accessing or using services, amongst others.
- Examples: Migrant women experience more discrimination and objectification, compared to non-migrant women, because of their immigration status.

1. **Recommendations**

- Includes information on recommendations to improve the system of care for migrant women.

1. **Missing code**

- This code will be used when no other codes are capturing what is being said. These codes will then be discussed and new codes, if necessary, will be created.
